# Therapie einer extremen Anämie mit vernetztem Rinderhämoglobin: Fallbericht mit Literaturüberblick

**DOI:** 10.1007/s00101-020-00864-w

**Published:** 2020-10-01

**Authors:** A. Meiser, H. Knoll, T. Meisel, M. Schröder, T. Volk

**Affiliations:** 1 Interdisziplinäre Operative Intensivstation, Klinik für Anästhesiologie, Intensivmedizin und Schmerztherapie, Universitätsklinikum des Saarlandes, Kirrberger Str. 100, 66424 Homburg/Saar, Deutschland; 2 Klinik für Allgemeine Chirurgie, Viszeral-, Gefäß- und Kinderchirurgie, Universitätsklinikum des Saarlandes, Kirrberger Str. 100, 66424 Homburg/Saar, Deutschland

**Keywords:** Polymerisiertes bovines Hämoglobin, Methämoglobin, Inhalative Sedierung, Isofluran, Polymerized bovine hemoglobin, Methemoglobin, Inhaled sedation, Isoflurane

## Abstract

Eine 29-jährige Zeugin Jehovas erlitt nach intrauterinem Fruchttod in der 25. Schwangerschaftswoche ein akutes Nierenversagen und eine schwere Anämie, bedingt durch Blutverlust und Hämolyse, erlitt. Wegen ihrer religiösen Überzeugung lehnte die Patientin Bluttransfusionen kategorisch ab. Trotz Umsetzung aller Empfehlungen des *Patient Blood Management* fiel der Hämoglobin(Hb)-Wert kontinuierlich. Am Tag 10 wurde sie bei Hb 1,9 g/dl plötzlich bewusstlos und musste intubiert und beatmet werden. Wegen organprotektiver Effekte und der guten Steuerbarkeit wurde sie inhalativ mit Isofluran sediert. Isofluran ermöglichte jeweils eine rasche neurologische Beurteilbarkeit in Sedierungsfenstern sowie eine regelmäßige, ruhige Spontanatmung bei tiefer Sedierung mit reduziertem Sauerstoffverbrauch. Als Ausdruck einer posthypoxischen Enzephalopathie zeigte sie in den Sedierungsfenstern Krampfanfälle, die im EEG bestätigt und antikonvulsiv behandelt wurden. Bei Hb 1,8 g/dl erhielt sie 2 Infusionsbeutel mit polymerisiertem bovinem Hämoglobin (Hemopure®, Fa. Hemoglobin Oxygen Therapeutics LLC, Souderton, PA, USA), wegen der kurzen Halbwertszeit mehrmals wiederholt an den Folgetagen. Eine beachtliche Methämoglobinämie wurde festgestellt. Auch nach Abzug des Met-Hb zeigten sich Hb-Anstiege um 0,4–0,8 g/dl nach den Gaben. Die Sauerstoffbindungskapazität des Blutes konnte damit initial um 33 % gesteigert werden. Es kam zu einer vollständigen neurologischen Erholung, und die Patientin wurde schließlich vom Respirator entwöhnt, jedoch weiterhin dialysepflichtig nach 38 Tagen in eine andere Klink verlegt.

Wenn die Gabe von Fremdblut keine Option darstellt, kann durch Gabe von polymerisiertem bovinem Hämoglobin als Ultima-Ratio-Therapie die Sauerstofftransportkapazität vorübergehend erhöht werden. Eine inhalative Sedierung mit Isofluran zur Senkung des Sauerstoffverbrauchs erscheint gleichermaßen vorteilhaft.

**Zusatzmaterial online:**

Die Online-Version dieses Beitrags (10.1007/s00101-020-00864-w) enthält eine Stellungnahme des Krankenhausinformationsdienstes zum fremdblutfreien Behandlungsmanagement von Jehovas Zeugen. Beitrag und Zusatzmaterial stehen Ihnen auf www.springermedizin.de zur Verfügung. Bitte geben Sie dort den Beitragstitel in die Suche ein, das Zusatzmaterial finden Sie beim Beitrag unter „Ergänzende Inhalte“.

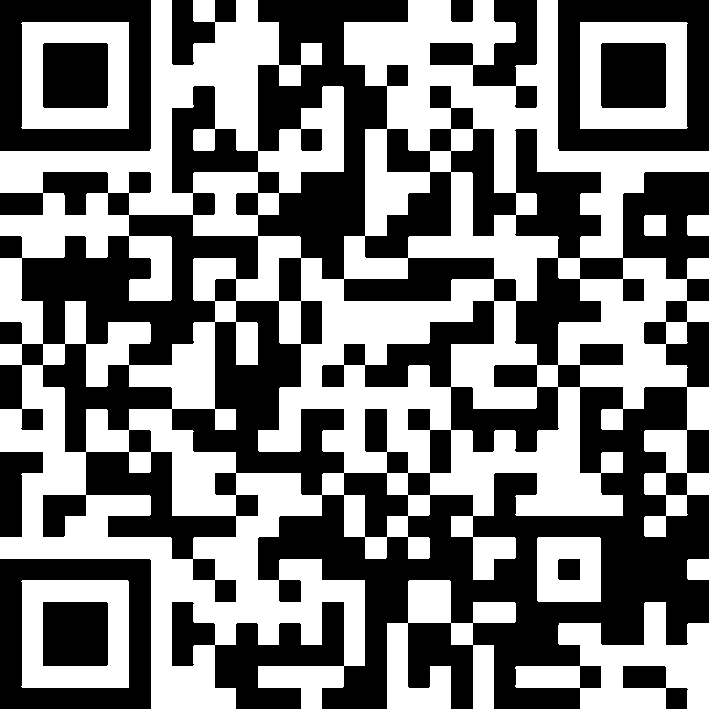

## Abstract (en)

This article reports the case of a 29-year-old female Jehovah’s Witness with severe anemia after intrauterine fetal death in the 25th week of gestation, complicated by vaginal bleeding, acute renal failure and hemolysis. Due to her religious beliefs the patient categorically refused blood transfusions. Despite adhering to the recommendations for patient blood management, the hemoglobin (Hb) level gradually decreased to 1.9 g/dl on day 10, when she fainted and had to be sedated and invasively ventilated. Inhalative isoflurane was chosen for sedation because of its potential organ-protective effects and because it provides deep sedation with reduced oxygen requirements, while enabling rapid neurological examination during the sedation windows as well as regular and calm spontaneous breathing. Posthypoxic encephalopathy was demonstrated clinically and electroencephalographically by seizure activity during the sedation windows. Anticonvulsive treatment was started. At a hemoglobin of 1.8 g/dl, she received 2 units of polymerized bovine hemoglobin (Hemopure®, Hemoglogin Oxygen Therapeutics LLC, Souderton, PA, USA), repeated several times on subsequent days because of its short half-life. Considerable methemoglobinemia was noted. After subtracting methemoglobin, the hemoglobin rose by 0.4–0.8 g/dl after each 2 units, initially increasing the oxygen binding capacity of the blood by 33%. After a full neurological recovery and weaning from the ventilator but still on hemodialysis, the patient was transferred to another hospital after 38 days.

If allogeneic blood transfusion is not an option, administration of polymerized bovine hemoglobin can temporarily increase the oxygen transport capacity as a last resort treatment. Reduction of oxygen requirements by deep inhaled sedation with isoflurane also seems beneficial and provides advantages.

## Anamnese

Eine 29-jährige, 172 cm große und 93 kg wiegende Zeugin Jehovas (ZJ) begab sich wegen vaginaler Blutungen in die stationäre Behandlung. Sie war in der 25. Woche schwanger, Primipara, Secundigravida. Bei intrauterinem Fruchttod wurden eine Geburtseinleitung und bei fortgesetzter Blutung eine Nachkürettage durchgeführt. Prä- und postoperativ wurde sie von Anästhesisten und Gynäkologen auf die medizinische Notwendigkeit einer Bluttransfusion hingewiesen, um einer Gefahr für Leib und Leben vorzubeugen. Aufgrund ihres Glaubens lehnte die Patientin die Gabe von Fremdblut sowie jeglichen anderen Blutprodukten ab. Etwa 18 h nach der Kürettage wurde die Patientin wegen Abfalls des Hämoglobins (Hb) auf 4,3 g/dl auf die Intensivstation aufgenommen. Bis dahin waren alle Vital- und Laborparameter bis auf Hb und Leukozyten unauffällig gewesen. Übelkeit, Kopf- und Oberbauchschmerzen wurden verneint. Vorerkrankungen waren nicht bekannt.

## Befund

Bei Aufnahme zeigte sich eine wache, voll orientierte, in Ruhe kreislaufstabile Patientin ohne Blutungszeichen. Weder klinisch noch sonographisch gab es Hinweise auf eine fortgesetzte Blutung; laborchemisch zeigten sich Zeichen einer akuten Hämolyse, Erhöhungen der Transaminasen sowie eine Thrombozytopenie (Tab. 1). Die Patientin war anurisch, Fragmentozyten waren nicht nachweisbar; Blutkulturen und Vaginalabstriche zeigten kein Keimwachstum; im Abdomen-CT konnten weder Blutungszeichen noch Perfusionsstörungen noch Verhalte nachgewiesen werden.

**Table 1:** 

	Prä-OP	Tag 1	Tag 10	Tag 12	Tag 14	Tag 20	Tag 38
*Blutdruck [mm* *Hg]*	140/80	142/68	131/60	121/66	150/70	160/85	143/80
*Herzfrequenz [/min]*	90	106	136	107	91	116	85
*Temperatur [°C]*	–	37,4	37	36,8	37,4	38,2	37,4
*Hämoglobin [g/dl]*	8,1	4,6	1,9	2,3	3,3	4,9	5,6
*Met-Hb (%)*	–	–	–	18	28	7	–
*Thrombozyten [10*^*9*^*/l]*	219	84	267	293	305	300	291
*Quick [%]*	102	77	73	65	78	81	80
*PTT [s]*	19	28	29	24	18	32	18
*Fibrinogen [mg/dl]*	312	232	296	315	361	549	253
*Haptoglobin [mg/dl]*	123	<5	<5	<5	6	<5	37
*ASAT [U/l]*	19	550	42	82	132	63	19
*ALAT [U/l]*	10	200	19	22	27	15	24
*GLDH [U/l]*	–	520	21	21	22	6	2
*LDH [U/l]*	231	2600	1514	1243	1281	902	424
*Kreatinin [mg/dl]*	0,48	3,3	(CVVHD)	(CVVHD)	(CVVHD)	(CVVHD)	(CVVHD)
*Leukozyten [10*^*9*^*/l]*	15,5	15	13	22	19	11	9
*CRP [mg/l]*	10	150	70	63	115	185	3
*PCT [ng/ml]*	–	5,5	1,6	1,4	1,6	1,6	0,25

Die präoperativen Parameter (*Prä-OP*) wurden nach dem Abort und vor der Nachkürettage, die Parameter am Tag 1 etwa 18 h später bei Aufnahme auf die Intensivstation erhoben

*Met-Hb* Methämoglobin (prozentualer Anteil am Gesamthämoglobin), *PTT* „partial thromboplastin time“, *ASAT* Aspartataminotransferase, *ALAT* Alaninaminotransferase, *GLDH* Glutamatdehydrogenase, *LDH* Laktatdehydrogenase, *CRP* C-reaktives Protein, *PCT* Prokalzitonin, *CVVHD* Dialysebehandlung („continuous venovenous haemodialysis“)

## Diagnose

Aufgrund dieser Befunde gingen wir von einer Anämie nach Totgeburt mit vaginaler Blutung sowie einem postpartalen HELLP(„hemolysis, elevated liver enzymes, low platelets“)-Syndrom mit Hämolyse, Leberenzymanstieg und Thrombozytenabfall aus. Als Ursache des Nierenversagens kamen zum einen die blutungsbedingte Anämie, die Hämolyse, oder eine septische Ursache bei deutlich erhöhten Entzündungsparametern infrage.

## Therapie

Trotz ausreichender Volumengabe blieb die Patientin anurisch. Nach ausführlicher Aufklärung der Patientin und der Angehörigen wurde eine Hämodialyse zur Therapie des Nierenversagens akzeptiert und als kontinuierliches Verfahren mit einer Zitratantikoagulation ab dem 3. Tag auf der Intensivstation (Tag 3) etabliert.

**Figure 1:**
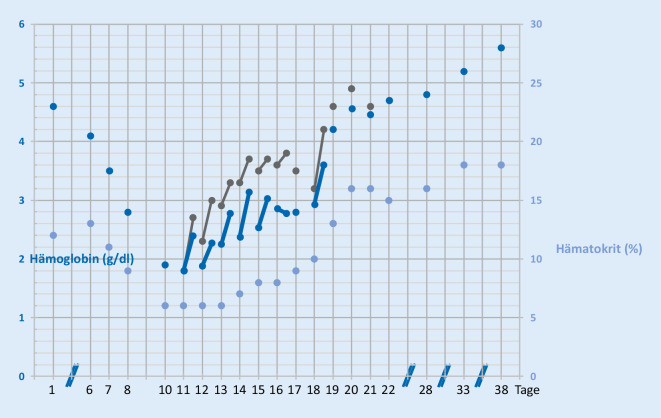

Alle Empfehlungen zum Patient Blood Management wurden umgesetzt (Infobox 1) [17]. Es erfolgten eine Gerinnungsoptimierung mit Tranexamsäure und Desmopressin sowie eine Behandlung der Hämolyse mit Prednisolon. Zur Stimulation der Blutbildung wurden 3‑mal wöchentlich Erythropoetin, anfangs 20.000 IE, zusammen mit einmalig 1000 mg Eisen, sowie täglich 1000 µg Cobalamin und 5 mg Folsäure i.v. verabreicht. Durch Atemphysiotherapie und Sauerstoffinsufflation wurde die Sauerstoffsättigung optimiert. Blutentnahmen wurden reduziert und pädiatrische Monovetten verwendet. Die Dialysebehandlung wurde unter strikter Antikoagulation mit Zitrat durchgeführt, um ein Stehenbleiben des Systems auf jeden Fall zu vermeiden. Beim Systemwechsel der Dialyse wurde darauf geachtet, jeweils das gesamte Blutvolumen zurückzugeben. Dennoch fiel der Hb-Wert kontinuierlich bis auf 1,8 g/dl am Tag 11 ab (Abb. 1).

## Der klinische Verlauf

Die Patientin wurde täglich in den Stand mobilisiert. Am Tag 8 (Hb 2,8 g/dl) gelang die Mobilisation nur bis an die Bettkante und musste wegen einer Tachykardie abgebrochen werden. Am Tag 10 (Hb 1,9 g/dl) wurde die Patientin während der ärztlichen Untersuchung in Rückenlage plötzlich bewusstlos und musste schutzintubiert werden. Zur Reduktion des Sauerstoffverbrauchs wurde die Patientin inhalativ mit Isofluran sediert. Wiederholtes Shivering, Pressen gegen die Beatmung und Strecksynergismen wurden als Krampanfälle gedeutet und mit Levetiracetam und Vertiefung der Sedierung behandelt. Ab Tag 15 (Hb 3,5 g/dl) atmete die Patientin spontan mit Druckunterstützung. Am Tag 17 (Hb 3,5 g/dl) wurden im Sedierungsfenster krampftypische Potenziale im EEG nachgewiesen, sodass wir von einer posthypoxischen Enzephalopathie ausgingen. Die antikonvulsive Medikation wurde erhöht. In Sedierungsfenstern ab Tag 20 (Hb 4,9 g/dl) zeigte die Patientin lediglich Hirnstammreflexe, jedoch keinerlei Großhirnaktivität. Eine kranielle Computertomographie am Tag 23 ergab keinen pathologischen Befund. Am Tag 24 wurde nach einer Punktionstracheotomie die inhalative Sedierung beendet; am Tag 25 reagierte sie verlangsamt auf Ansprache; am Tag 26 war eine gezielte Kommunikation durch Mimik, Kopfnicken und -schütteln möglich, am Tag 29 wurde sie erstmals wieder an die Bettkante mobilisiert; am Tag 34 war sie nahezu vollständig vom Beatmungsgerät entwöhnt, und am Tag 38 (Hb 5,6 g/dl) wurde sie – neurologisch unauffällig, jedoch weiterhin dialysepflichtig – auf eigenen Wunsch in eine andere Klinik verlegt.

## Polymerisiertes bovines Hämoglobin als Blutersatz (Hemopure®)

Nach dem Bewusstseinsverlust am Tag 10 trotz Ausschöpfung aller Therapiemaßnahmen gingen wir von einer akuten Gefährdung der jungen Mutter aus. In wiederholten Gesprächen mit Mitgliedern der Glaubensgemeinschaft, dem vorsorgebevollmächtigten Ehemann und der Patientin auch unter vier Augen war die Gabe von Fremdblut stets abgelehnt worden. Gemeinsam mit der Glaubensgemeinschaft wurde diskutiert, das Blutersatzpräparat Hemopure® (Fa. Hemoglobin Oxygen Therapeutics LLC, Souderton, PA, USA) einzusetzen, welches in keinem Land der europäischen Union zugelassen ist.

Hemopure® besteht aus vernetztem Rinder-Hb. Das Polymer mit einer molaren Masse von 250 kg/mol ist in 250 ml Ringer-Lösung suspendiert. Ein Infusionsbeutel enthält 32 g Hb mit einer Sauerstoffbindungskapazität von 1,39 ml O_2_/g Hb, analog der des menschlichen Hb. Durch eine Rechtsverschiebung der Sauerstoffbindungskurve ist die Sauerstoffabgabe an die Gewebe verbessert, die Sauerstoffaufnahme in der Lunge bei hohen Sauerstoffkonzentrationen jedoch nicht verschlechtert. Die Lösung zeigt kolloidale Eigenschaften, sodass die Gefahr einer Volumenüberladung besteht. Da es sich um eine zellfreie Lösung handelt, ist das immunisierende Potenzial gering, eine serologische Verträglichkeitsprobe nicht durchführbar. Das polymerisierte Hb wird in der Niere nicht filtriert und vom retikuloendothelialen System rasch abgebaut, sodass die Gabe täglich wiederholt werden muss. Es ist nicht wie das körpereigene Hb durch die enzymatische Ausstattung der Erythrozyten vor Oxidation geschützt und neigt daher zur Bildung von Methämoglobin (Met-Hb), welches für den Sauerstofftransport nicht mehr zur Verfügung steht. Die zeitgleiche Infusion von Ascorbinsäure als Antioxidans wird daher empfohlen. Hemopure ist ohne Kühlung 3 Jahre haltbar, wird in den USA hergestellt und ist in Russland und Südafrika als Blutersatzmittel zugelassen [16].

Der bevollmächtigte Ehemann wurde über die Anwendung des nichtzugelassenen Arzneimittels als individuellen Heilversuch aufgeklärt und um schriftliches Einverständnis gebeten. Hemopure® wurde bei der Firma bestellt und sollte aus den USA oder aus Südafrika geliefert werden. Dies hätte jedoch 7 bis 10 Tage in Anspruch genommen. In der Notfallsituation wurde ein Vorrat an Studienmedikation aus dem benachbarten Ausland vom Hersteller und vom Studienzentrum freigegeben und über Nacht geliefert.

**Figure 2:**
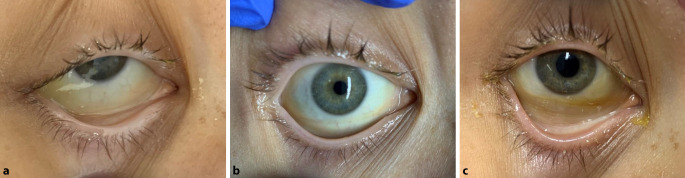

An den Tagen 11 bis 16 und am Tag 18 wurden jeweils 2 Infusionsbeutel à 250 ml über je 2 h nacheinander infundiert. Zuvor und danach wurden Hb und Met-Hb der Patientin bestimmt (Abb. 1). Trotz Gabe von Ascorbinsäure [18] und Methylenblau [5] ließ sich die Bildung von Met-Hb bis über 20 % des Gesamt-Hb nicht verhindern. Hierunter war die pulsoxymetrische Sättigung klinisch nicht mehr verwertbar und zeigte Werte unter 80 % an. Auch wenn man das Met-Hb, welches keinen Sauerstoff transportieren kann, vom Gesamt-Hb abzieht, kam es zu einem messbaren Hb-Anstieg unter der Therapie, der nicht durch patienteneigenes Hb erklärt werden kann, da der Hämatokrit vom Tag 10 bis Tag 13 gleichblieb und auch danach nur sehr viel langsamer anstieg. Klinisch zeigte sich ein Sklerenikterus, welcher durch Ablagerungen von Hemopure®-Abbauprodukten erklärbar ist (Abb. 2). Das Gesamtbilirubin stieg unter der Therapie bis maximal 2,9 mg/dl an Tag 15.

## Diskussion

Wir berichten den Fall einer jungen Zeugin Jehovas, die einen kontinuierlichen Hb-Abfall bis auf 1,8 g/dl ohne neurologische Schäden überlebt hat. Entscheidende Therapiemaßnahmen waren neben Stimulation der Erythropoese und Behandlung der Hämolyse die Senkung des Sauerstoffverbrauchs durch tiefe, inhalative Sedierung mit Isofluran sowie die Gabe des Blutersatzstoffes Hemopure®.

Etliche Fallberichte beschreiben eine erfolgreiche Therapie extrem niedriger Hb-Werte bei jungen (maximales Alter 44 Jahre), kardiovaskulär gesunden ZJ, einige davon aus Europa [4, 21, 22], die meisten aus den USA [5, 7, 10, 20, 23, 24, 28]. Sowohl akute postpartale [21, 23, 24], postoperative [7, 22, 28] oder posttraumatische Anämien [20] als auch langsame Hb-Abfälle bei hämolytischer Anämie [10] oder Chemotherapie bei Leukämien [4, 5] wurden überlebt. Die niedrigsten dokumentierten Hb-Werte reichen von 3,9 bis 1,3 mg/dl [4] sowie in einem Fall „nicht mehr messbar“ bei einem Hämatokrit von 3 % [23].

In fast allen Fällen wurde dabei hochdosiertes Erythropoetin eingesetzt, aber nicht immer konnte damit eine rasche Erythropoese erzielt werden. Als Gründe werden Infekte, Nebenwirkung der Chemotherapie, aber auch die anämiebedingte Hypoxie des Knochenmarks selbst angeführt. In einem Fall konnten Kreislaufparameter (Laktacidose, zentralvenöse Sättigung und Herzfrequenz) durch Erhöhung der F_I_O_2_ auf 100 % verbessert werden [23], in einem anderen Fall zeigten wiederholte Zyklen einer hyperbaren Sauerstofftherapie keinen messbaren Effekt [24].

In den USA wurden in 5 publizierten Fällen 2 bis 15 Infusionsbeutel Hemopure® eingesetzt [5, 7, 10, 20, 24]. Die Autoren beschreiben messbare Hb-Anstiege, aber auch eine Erhöhung des Met-Hb. Über die Anwendung von Hemopure® in Ländern der Europäischen Union wurde bislang nicht berichtet.

Fallberichte zeigen einen Publikationsbias, da oftmals nur ein gutes Outcome publiziert wird. Das Mortalitäts- und Morbiditätsrisiko extremer Anämien kann nur durch systematische Analysen abgeschätzt werden. Im Rahmen einer retrospektiven Analyse wurde von einem postpartalen Todesfall einer 40-jährigen ZJ mit einem Hb von 3,4 g/dl berichtet [12]. Bei einer nationalen Registeranalyse in den Niederlanden wurden 6 postpartal verstorbene ZJ auf 8850 Geburten identifiziert, welches einer 6‑fach erhöhten Müttersterblichkeit entspricht [27]. Die Mortalität von 300 Patienten, die Bluttransfusionen ablehnten, in den USA an spezialisierten Zentren operiert wurden und die postoperative Hb-Werte kleiner 8 g/dl aufwiesen, korrelierte mit dem niedrigsten gemessenen Hb-Wert. Sie betrug 0 % für Hb-Werte 7–8 g/dl und stieg bis auf 54 % und 100 % für Hb-Werte 2–3 sowie 1–2 g/dl [2].

In den meisten Fallberichten wurden Patienten mit extremer Anämie sediert und beatmet. Wir verwenden für die tiefe Sedierung Isofluran, verabreicht über das AnaConDa®-System (Fa. Sedana Medical, Danderyd, Schweden). Hierbei handelt es sich um einen „off label use“, der jedoch durch zahlreiche randomisierte Studien (reviewed in [8, 14]) und mehrere nationale Leitlinien gedeckt ist [1, 3, 26]. Die rasche neurologische Beurteilbarkeit [6] in Sedierungsfenstern erlaubte uns die Diagnose einer posthypoxischen Enzephalopathie durch Nachweis epileptiformer Potenziale im EEG sowie die Initiierung einer ruhigen Spontanatmung [13, 15], um Zwerchfellatrophie und Atelektasenbildung in den dorsobasalen Lungenabschnitten vorzubeugen, bei zugleich tiefer Sedierung mit reduziertem Sauerstoffverbrauch. Des Weiteren werden volatilen Anästhetika organprotektive Wirkungen zugeschrieben (reviewed in [9]).

In einer Metaanalyse von 16 Studien mit insgesamt 3711 Patienten, die verschiedene zellfreie hämoglobinbasierte Blutersatzstoffe mit der Gabe von Erythrozyten verglichen, zeigten sich eine höhere Mortalität und ein häufigeres Auftreten von Myokardinfarkten in den Studiengruppen [19]. Ursächlich ist vermutlich ein Abfangen von Stickstoffmonoxid als potentem körpereigenem Vasodilatator [11]. Aufgrund dieser Daten wurde keines der Präparate von der US-amerikanischen* Food and Drug Administration* (FDA) zugelassen. Wenn Erythrozytenkonzentrate jedoch keine Option darstellen, ist das Risiko, an einer extremen Anämie zu versterben, wesentlich höher als das gering erhöhte Mortalitätsrisiko durch Blutersatzstoffe [25].

Der plötzliche Bewusstseinsverlust und die nachgewiesenen Krampfpotenziale bei einer Patientin ohne Epilepsie in der Vorgeschichte beweisen, dass die Sauerstoffversorgung des Gehirns zeitweise kompromittiert war. Wir gehen daher davon aus, dass sich die Gabe von Hemopure® auf den weiteren Verlauf positiv ausgewirkt hat, auch wenn die effektive, Met-Hb-bereinigte Hb-Wert-Steigerung nur 0,4–0,8 g/dl ausmachte. Dies entsprach jedoch initial einer Steigerung der Sauerstoffbindungskapazität des Blutes um 33 %.

Bei einem Hb von 1,8 g/dl und einem geschätzten Blutvolumen von 6,3 l (0,07 * 90 kgKG) kann das Hämoglobin der Patientin etwa 158 ml O_2_ binden. Zusätzlich sind bei einem p_a_O_2_ von 250 mm Hg etwa 50 ml O_2_ physikalisch gelöst. Eine Einheit Hämopure mit 32 g Hb kann 44 ml O_2_ binden. Durch 2 Einheiten des Präparats konnte also der Sauerstoffgehalt des gesamten Blutes um 42 % auf 296 ml O_2_ gesteigert werden. Allerdings muss erwähnt werden, dass ein Teil des zugeführten Hämoglobins trotz Gabe von Ascorbinsäure (und später zusätzlich Methylenblau) sehr rasch zu Met-Hb oxidierte und damit keinen Sauerstoff mehr transportieren konnte. In Abb. 1 ist daher der jeweilige Anstieg des Hb unter der täglich wiederholten Hemopure®-Therapie sowohl als Gesamt-Hb wie auch als Anteil ohne Met-Hb dargestellt. Der rasche Abbau durch das retikuloendotheliale System führte dazu, dass bereits am nächsten Morgen das Gesamt-Hb wieder von 2,7 auf 2,3 (Anteil ohne Met-Hb: von 2,5 auf 1,9) g/dl abgefallen war.

Es ist einem Zufall zu verdanken, dass das Arzneimittel kurzfristig aus dem nahen Ausland besorgt werden konnte. Dies hat der Patientin wahrscheinlich das Leben gerettet. In den USA ist Ärzten die Verabreichung nichtzugelassener Medikamente als „compassionate use“ nur erlaubt mit einer Genehmigung durch die FDA, die für ZJ bislang in 109 Fällen erteilt wurde (zitiert aus [25]). In Deutschland kann ein Arzt im Sinne eines Heilversuchs im Einzelfall nichtzugelassene Arzneimittel ohne behördliche Genehmigung unter Berufung auf einen rechtfertigenden Notstand einsetzen. Dabei trägt der Arzt die Verantwortung; er muss umfassend aufklären, ein Einverständnis einholen und die Behandlung sorgfältig dokumentieren [29]. Für einen wiederholten Einsatz bei mehreren Patienten mit der gleichen Indikation kann ein Arzneimittelhärtefallprogramm beim Bundesamt für Arzneimittel und Medizinprodukte beantragt werden [30]. Allerdings stellt die Beschaffung eines Medikaments aus dem Ausland letztlich eine zeitaufwendige Prozedur dar, da sowohl eine Ausfuhr- als auch eine Einfuhrgenehmigung beschafft werden müssen. Diese bürokratischen Hürden nehmen einige Tage bis Wochen in Anspruch, selbst wenn der Antrag als „medizinischer Notfall“ gekennzeichnet ist.

### Infobox 1 Patient Blood Management^a^

- Sorgfältige Blutstillung
- Optimierung der Hämostase
  - Physiologische Rahmenbedingungen (Temperatur, Kalzium, pH)
  - Antifibrinolytika (Tranexamsäure 1000 mg)
  - Thrombozytenfunktion (Desmopressin 0,3 µg/kgKG)
- Therapie einer Hämolyse (z. B. Prednisolon 1 mg/kgKG)
- Therapie der Anämie
  - Substitution von Eisen
  - Substitution von Folsäure und Vitamin B_12_
  - Erythropoetin, hochdosiert
- Maximierung der Oxygenierung des Blutes
  - Erhöhte F_I_O_2_
  - Prophylaxe von Atelektasen
- Reduktion des Sauerstoffbedarfs
  - Bettruhe
  - Gegebenenfalls Sedierung und Beatmung
- Reduzierte Blutentnahmen
  - Reduktion der Anzahl auf das notwendige Minimum
  - Reduktion des Volumens pro Entnahme: *Verwendung pädiatrischer Monovetten; geringere Füllung der Monovetten, falls dies die Analyse nicht verfälscht*
  - Verwendung geschlossener Entnahmesysteme zur Vermeidung des Verwerfens von Blutresten

^a^Modifiziert nach Meybohm et al. [17]

## Fazit für die Praxis

Wenn die Gabe von Fremdblut aus medizinischen (Autoimmunhämolyse), logistischen (Verfügbarkeit) oder aus Glaubensgründen keine Option darstellt, kann durch Gabe von polymerisiertem bovinem Hämoglobin als Ultima-Ratio-Therapie die Sauerstofftransportkapazität vorübergehend erhöht werden. Wegen der kurzen Halbwertszeit sind wiederholte Gaben erforderlich, zumal eine rasche Methämoglobinbildung den Sauerstofftransport des zugeführten Hämoglobins kompromittiert. Eine Senkung des Sauerstoffverbrauchs ist gleichermaßen wichtig; eine inhalative Sedierung mit Isofluran stellt hier eine gute Option dar.

## Caption Electronic Supplementary Material


