# Supplementary material for: Treatment of extreme anemia with polymerized bovine hemoglobin: Case report and review of the literature
Source: Anaesthesist. 2020 Oct 1;70(4):308–15. [Article in German] doi: 10.1007/s00101-020-00864-w (PMC8026446; doi:10.1007/s00101-020-00864-w)
Supplement: Supplementary file 1 [file 101_2020_864_MOESM1_ESM.pdf]

Zusatzmaterial zum Beitrag

Meiser A., Knoll H., Meisel T. et al. (2020) Therapie einer extremen Anämie mit vernetztem Rinder-Hämoglobin. Fallbericht und Literaturüberblick. *Der Anaesthesist*

Beitrag und Zusatzmaterial stehen Ihnen auf www.springermedizin.de zur Verfügung. Bitte geben Sie dort den Beitragstitel „Therapie einer extremen Anämie mit vernetztem Rinder-Hämoglobin“ in die Suche ein.

Fremdblutfreies Behandlungsmanagement von Jehovas Zeugen

Andreas Hattinger*, Simon von Arx*

* Krankenhausinformationsdienst für Zeugen Jehovas, Bereich D-A-CH-Region und Luxemburg

Jehovas Zeugen sind bei Ärzten dafür bekannt, allogene Bluttransfusionen abzulehnen. Das schließt Vollblut, Erythrozyten, Leukozyten, Thrombozyten und Plasma ein. Auch die präoperative Eigenblutspende wird abgelehnt. Ihre Haltung basiert auf mehreren Bibelpassagen, z.B. „Enthaltet euch ... von Blut“ (Apostelgeschichte 15:29).[1]

Obwohl die Ablehnung von allogenen Bluttransfusionen eine grundlegende Glaubenslehre von Jehovas Zeugen ist, sind einige damit verwandte Therapieoptionen eine persönliche Entscheidung. Deshalb müssen Ärzte im Aufklärungsgespräch immer den individuellen Patientenwunsch ermitteln. Dazu gehört die Verwendung von Eigenblut im Rahmen eines medizinischen Verfahrens oder einer Therapie, wie maschinelle Autotransfusion, kardiopulmonaler Bypass, Nierenersatztherapie oder therapeutische Apherese unter Verwendung von Plasma-Alternativen.[2] (Extrakorporale Kreisläufe dürfen allerdings nicht mit allogenem Blut in Betrieb genommen werden.) Dazu gehört auch der Einsatz von Gerinnungsfaktorkonzentraten (z.B. Einzelfaktor- oder Prothrombinkomplexkonzentraten), Albumin, Immunglobulinen und hämoglobinbasierten Sauerstoffträgern (unabhängig davon, ob sie menschlichen oder tierischen Ursprungs sind), wie im Fallbericht von Meiser et al. in der vorliegenden Ausgabe von *Der Anaesthesist* beschrieben.[3]

Korrespondenzadresse:
Andreas Hattinger
Krankenhausinformationsdienst für Zeugen Jehovas
Am Steinfels 1; 65618 Selters/Taunus
Tel.: +49 6483 41-2818
E-Mail: hid.de@jw.org

Offenlegung: Die Autoren erklären, dass keine Interessenkonflikte vorliegen.

[1] https://www.jw.org/de/biblische-lehren/fragen/bibel-zum-thema-bluttransfusion/; abgerufen am 12.07.2020
[2] Habler O, Voss B Perioperatives Management bei Zeugen Jehovas. Spezielle Berücksichtigung der religiös motivierten Ablehnung von Fremdblut. *Anaesthesist* 2010;59(4):297-311.
[3] Meiser A, Knoll H, Meisel T, Schröder M, Volk T. Treatment of extreme anemia with polymerized bovine hemoglobin. *Anaesthesist* 2020. Im Druck.

Draft
